# Electrospun Polycaprolactone (PCL) Nanofibers Induce Elongation and Alignment of Co-Cultured Primary Cortical Astrocytes and Neurons

**DOI:** 10.3390/mi16030256

**Published:** 2025-02-25

**Authors:** Kayleigh Nutt, Zoe Dombros-Ryan, Ruxandra Birea, Emily Victoria Franks, Sarah Eastham, Morgan Godwin, Chris F. Adams, Divya Maitreyi Chari, Stuart Iain Jenkins

**Affiliations:** 1 Neural Tissue Engineering: Keele (NTEK), Keele University, Keele ST5 5BG, UKx3f51@students.keele.ac.uk (Z.D.-R.); e.v.franks@keele.ac.uk (E.V.F.); c.adams@keele.ac.uk (C.F.A.); 2 School of Life Sciences, Keele University, Keele ST5 5BG, UK; 3 School of Medicine, Keele University, Keele ST5 5BG, UK; 4 Department of Biomedical Engineering, University of Strathclyde, Glasgow G4 0NW, UK

**Keywords:** microfibers, nanofibers, brain, biomaterials, polymers, electrospinning, neural tissue engineering, regenerative medicine, scaffolds, orientation

## Abstract

Neuromimetic in vitro models, simulating in vivo architecture/organization, are urgently needed to reduce experimental reliance on live animals. Our group recently reported a novel brain tissue derivation protocol, simultaneously deriving all major cortical cell types (including immune cells) in a facile protocol, generating a network of neurons in a single growth medium, which was interfaced with nanomaterials. This represents a significant advance, as tissue engineers overwhelmingly use diverse methods to derive and combine individual brain cells for materials-interfacing. However, this multicellular model lacked cellular directionality/structural organization (unlike the highly organized cortical circuits in vivo). Synthetic nanofiber constructs are of high value in tissue engineering, providing directional cues for cells. Most neuro-nanofiber studies employ simple monocultures of astrocytes/neurons and commonly use peripheral neurons rather than central nervous system populations. Here, we have interfaced our complex brain model (neurons/astrocytes derived simultaneously) with randomly oriented or aligned polycaprolactone (PCL) fiber meshes. Both cell types showed targeted extension along aligned fibers versus coverslips or random fibers. A new analysis method developed in-house demonstrated that peak orientations for astrocytes and neurons correlated with aligned nanofibers. Our data support the concept that nanofiber scaffolds can achieve organized growth of mixed cortical neural cell populations, mimicking neural architecture.

## 1. Introduction

Neuroregenerative research requires representative models of central nervous system (CNS) tissue and pathology. However, in vitro models often lack key features of CNS cytoarchitecture and/or are technically complex or expensive. It has recently been demonstrated that it is feasible for all major brain cell types (including immune cells) to be co-cultured in a single growth medium using a simple technical protocol [1]. This method overcomes several limitations associated with the use of multiple, separate protocols to derive and combine neural cells to mimic the complex multicellular nature of brain tissue. Hallmark pathological responses to injury occurred in this model, demonstrating its utility as a testbed for neurological therapies. This mixed cell model is low cost, adaptable for a range of analyses, and easily accessible for researchers. Further, the model has the potential to reduce or partially replace animal use in neurotrauma research, in line with the goals of the 3Rs (to reduce, refine and replace animal usage) [2,3].

Although all major neural cell types are present within this model, cellular arrangement is random and disorganized, unlike the developing cortical tissue from which the cells were derived. During development, cortical tissue is highly stereotypical, with aligned cytoarchitecture: long cell processes, in parallel, spanning the multiple layers of the cortex [4]. Without directional guidance cues, dissociated cortical cells are limited in their capacity to replicate this cytoarchitecture.

As a potential solution, electrospun nanofibers have been shown to act as effective guidance cues for neural cell orientation and process extension. This offers utility for simulating CNS cytoarchitecture in vitro through precise spatial/topographical control of cell growth. Fibers can be synthesized by solution electrospinning [5], with tunable physicochemical properties (tensile strength, porosity, fiber diameter, etc.). Fibers can be layered at various densities, may be functionalized to simulate the extracellular matrix or to deliver sustained release of drugs, and/or serve as vehicles for cell transplantation [6,7,8,9,10,11,12]. Among the synthetic polymers most suited for electrospinning are polylactic acid (PLA) and polycaprolactone (PCL) [10,13,14,15,16,17,18]. Both are used clinically and have been tested in multiple clinical trials [13,18,19].

For neural cell culture, nanofibers are most frequently used with peripheral nervous system (PNS) axons or cells at the CNS-PNS interface, such as dorsal root ganglion (DRG) cells [16,20,21,22,23]. Numerous studies have also demonstrated neural stem cells and their progeny could align along nanofibers [24,25,26,27]. Whilst useful and demonstrating that neurites can be directed along nanofibers for considerable distances, it is unclear whether the same technique would induce alignment and elongation of *mixed cortical cell populations*. Of note, Bourke et al. reported that DRG (embryonic day 18) neurons consistently followed aligned fibers, but they also showed that hippocampal (HPC) neurites exhibited *bimodal* peaks of orientation, one peak indicating neurites matching the dominant fiber direction, the other indicating additional neurites extending approximately perpendicularly to fibers (aligned PCL fibers, laminin-coated, diameter 458 ± 209 nm) [28]. This observation raises the possibility of differing cell–fiber interactions for cells derived from different CNS regions, highlighting the importance of specifically assessing *cortical* cell cultures on nanofiber scaffolds.

A technique for generating a high degree of cortical cell alignment on fibers has been demonstrated previously [29,30]. This showed fine spatial control of glial alignment on PLA fibers. However, various challenges were encountered, including ‘leaking’ of cells through the construct onto the base of the culture well (not being contained within the final construct). Collagen hydrogels were incorporated to counter this, but the overall process then became time-consuming and technically challenging. Also, although this model included multiple glial cell types, it lacked neurons, limiting its neuromimetic capacity. Other studies have used primary cortical cells on fibers, but either without fiber alignment [25] or without generating a high degree of cell/process alignment [31].

Therefore, the development of a simple protocol, wherein multiple neural cell types can be simultaneously derived and interfaced with nanofiber scaffolds within a single protocol, is of high benefit for neuroregenerative research. Here, we have evaluated the feasibility of simultaneously growing mixed neuron–glia primary cortical populations on a commercially available mesh of PCL nanofibers with the application of a directionality assay to assess scaffold-induced neural cell alignment.

## 2. Materials and Methods

### 2.1. Materials

Unless otherwise stated, tissue culture-grade plastics, media, and media supplements were from Fisher Scientific (Loughborough, UK) and Sigma-Aldrich (Poole, UK). DAPI mounting medium was from Vector Laboratories (Peterborough, UK). Secondary antibodies were from Jackson ImmunoResearch Laboratories Inc. (West Grove, PA, USA).

Nanofiber constructs were purchased in both randomly oriented and aligned formats, ~21 mm diameter discs (Random: Z694517-12EA; Aligned: Z694614-12EA; manufactured by Nanofiber Solutions, Dublin, Ireland; supplied by Merck, London, UK). They were sterile in original packaging (electron beam irradiated). PCL as a bulk material is approved for clinical use, including drug delivery and as scaffolds for tissue remodeling [13,18,19]. These scaffolds have previously been used for cell culture, demonstrating lack of toxicity and capacity for cell association with these fibers [32,33,34].

### 2.2. Characterization of Nanofibers

Micrographs of nanofibers were taken using phase contrast and fluorescence microscopy (Evos XL Core microscope; Zeiss Axio Observer Z-stack microscope, Zen software v3.3.89, Wilmington, DE, USA). For scanning electron microscopy (SEM), sections of nanofiber constructs (prepared as per culture protocol) were gently pressed onto carbon stubs and then imaged (TM4000Plus Scanning Electron microscope, Hitachi High-Tech Europe, GmbH, Krefeld, Germany, accelerating voltage 10–20 kV).

### 2.3. Nanofiber Construct Characterization

**Figure 1:**
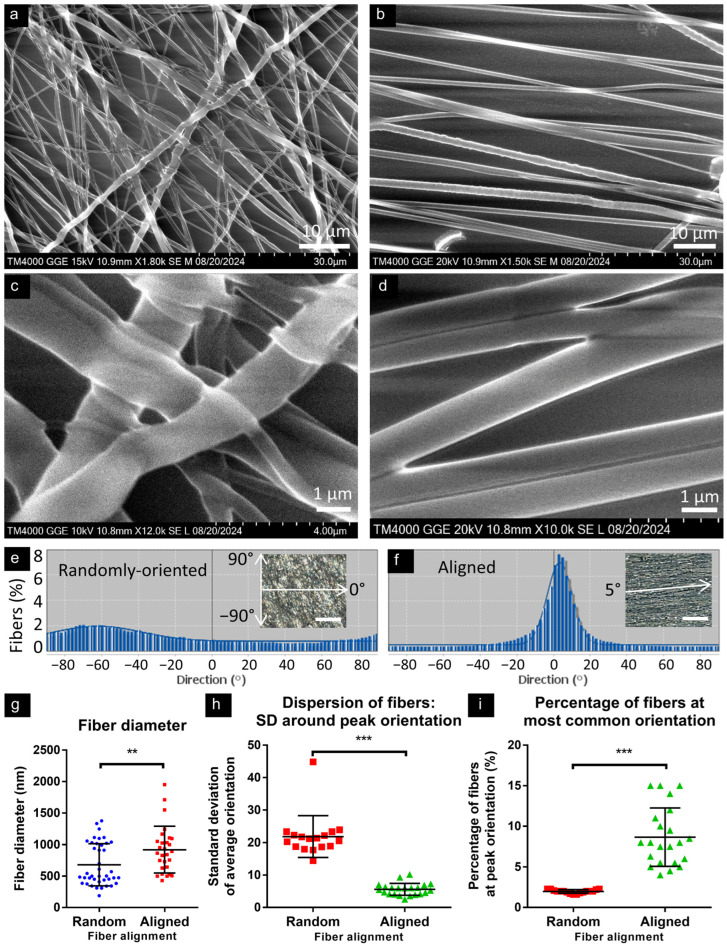
Diameter and orientation of polycaprolactone fiber constructs. Scanning electron micrographs of randomly oriented (**a**) and aligned (**b**) PCL nanofiber constructs. (**c**,**d**) Greater magnification of random and aligned fibers shows occasional adherence between fibers and overlapping/layering of fibers. Representative directionality histograms show no more than 2% of fibers sharing 1° of orientation in a random construct (**e**), whereas an aligned construct (**f**) had a strong peak, with 8% of fibers at peak orientation, with Gaussian distribution around this peak (insets show analyzed phase micrographs; scale bars: 50 µm). (**g**) Graph shows average fiber diameter (** *p* = 0.0063, unpaired two-tailed Mann–Whitney test, n = 40 (random) and 28 (aligned) fibers). (**h**) Random constructs show a wider distribution of fibers across orientations, illustrated by larger standard deviation (SD) around the most common orientation, vs. aligned constructs, which show small SD, i.e., narrow peak (compare with (**e**,**f**)). (**i**) Random constructs show few fibers at ‘peak’ direction, whereas aligned constructs show a greater percentage of fibers at peak orientation. For (**h**,**i**): *** *p * < 0.0001, unpaired two-tailed Mann–Whitney test, n = 17 (random) and 22 (aligned) micrographs.

Fibers were readily visualized through both light and electron microscopy and were clearly identifiable as either randomly oriented or (highly) aligned at all magnifications (Figure 1a–d). Directionality assessments were consistent with the construct descriptions as random and aligned orientations, with aligned constructs exhibiting clear peaks on histograms (Figure 1e,f). Fiber diameters were measured in electron micrographs (random: mean 681 ± 335 nm; aligned: 919 ± 371 nm; Figure 1g). Both formats contained nanofibers and microfibers, with random constructs having a greater prevalence of fibers with a diameter <500 nm (48% vs. 11%). The dispersion of fibers (range of fiber orientations) was greater in random constructs (Figure 1h), and the magnitude of the peak (highest percentage of fibers at a single orientation) was greater in aligned constructs (Figure 1i).

### 2.4. Preparation of Fiber Constructs and Glass Coverslips for Cell Culture

PCL fiber constructs were removed from packaging inside a laminar flow hood, placed in open Petri dishes, exposed to UV-C light (30 min), and then cut into smaller pieces. Constructs were held using forceps then cut into 1/8th (45°) wedges using fine, sharp dissecting scissors. These were then coated with polyornithine (30 min, RT), washed (distilled H_2_O: dH_2_O), coated with laminin (60 min, RT), then washed (PBS) and allowed to dry overnight within 24 well plates. Glass coverslips (13 mm) were sterilized within 24-well plates (70% isopropyl alcohol) then coated identically to fibers (polyornithine and laminin). Neurons will not readily adhere to uncoated glass, and laminin is widely used to promote adhesion. Polyornithine is widely used to enhance adherence of laminin to glass, especially for neuronal culture [35]. As this coating was used for control cultures, it was also applied to the fibers.

### 2.5. Primary Mouse Neuronal–Glial Co-Culture

Fresh tissues were dissected from mouse pups (CD-1), with litters ranging from eight to 12 pups. Keele University retains Home Office licensed authority as a designated premises, providing regulatory compliance for the care and welfare of the animals used in this study [Keele University Establishment license number: X350251A8 (copy available on request)]. Ethical approval for the Schedule 1 usage of the animals used in this study was obtained from the Keele University Animal Welfare and Ethical Review Body in 2017. Mice maintaining specified pathogen-free health status were housed and bred in the Keele Biological Service Unit, in accordance with the Code of Practice for the Housing and Care of Animals Bred, Supplied or Used for Scientific Purposes. Litters were maintained on a continuous 12:12 light cycle, 22.5 ± 0.4 °C, and 46% ± 5% humidity. Mice were bred and maintained according to the UK Code of Practice for the housing and care of animals used for scientific procedures, Animals (Scientific Procedures) Act 1986. Pups of both sexes were used in the study and culled via the Schedule 1 method of an overdose of anesthetic, sodium pentobarbital (Animalcare Ltd., York, UK), 1 mL/kg intraperitoneal injection, on postnatal day 0–1, weight ca. 1.5–3 g. Brains were dissected and transferred to a dissection medium (2.5% HEPES in Earle’s balanced salt solution) on ice.

Primary neuronal–glial cultures were prepared from dissociated cerebral cortices. In brief, cortices were isolated from whole brain (maintained in ice-cold PBS), then mechanically dissociated by scalpel in a Petri dish, and then trypsin-EDTA and DNase were added (125:62.5 µL per brain) with gentle trituration. This solution was placed on a rotary shaker (60 rpm; 37 °C; 5 min), then gently triturated, and returned to the shaker for a further 5 min. Fetal bovine serum (FBS; 1 mL) was added to interrupt enzyme action, the tube was immediately centrifuged (1200 rpm; 5 min), supernatant discarded, and pelleted cells resuspended in fresh NbB27 medium (Neurobasal-A supplemented with 2% B27, and final concentrations of glutamine 2 mM, penicillin 50 U mL^−1^, and streptomycin 50 µg mL^−1^). Cells were then counted and seeded on glass coverslips, pre-coated with polyornithine and laminin, as per Section 2.4.

### 2.6. Seeding Cells onto Nanofiber Constructs

Initial experiments seeded onto freshly washed fibers, and these were more prone to cells dispersing off the fibers onto the underlying culture plastic (this being still wet). With the culture plate and fibers dry, a small droplet of media more reliably remained on the fibers, increasing the number of cells adhering. For the 1/8th of a 2 cm construct used here, a 70 µL droplet was effective. At a concentration of 8 × 10^5^ cells/mL and an approximate construct area of 43 mm^2^ (1/8th of a 21 mm disc), cells were seeded at approximately 5.6 × 10^4^ cells per construct (~1300 per mm^2^). After allowing 30–60 min for cell adherence, wells were flooded with NbB27 medium (500 µL; incubated at 37 °C, 5% CO_2_:95% humidified air; 50% medium changes every 2–3 d). Coverslips (identically-coated with polyornithine and laminin) were seeded in parallel (70 µL cells; later flooded with 500 µL of medium), to allow comparison of cell morphologies.

### 2.7. Fixation and Immunostaining of Cultures

At 5 or 9 days in vitro (DIV), cultures were washed with phosphate buffered saline (PBS) then fixed using 4% paraformaldehyde in PBS (20 min; room temperature, RT). Fixed cells were incubated with blocker (5% normal donkey serum in PBS, 0.3% Triton X-100; RT; 30 min), then primary antibody in blocker [4 °C; overnight; rabbit anti-glial fibrillary protein (GFAP), from Agilent, Dako, Z0334, 1:500; mouse anti-BIII tubulin (TUBB3; clone Tuj1), from Biolegend (San Diego, CA, USA), 801202, 1:1000].

Cells were then washed (PBS), incubated with blocker (RT; 30 min), and incubated with fluorescein isothiocyanate (FITC)- or cyanine3 (Cy3)-conjugated secondary antibody in blocker (1:200; RT; 2 h). Finally, coverslips were washed (PBS) and mounted with the nuclear stain DAPI (Vectorshield mounting medium, Vector Laboratories, Peterborough, UK).

### 2.8. Fiber Diameter and Directionality Analyses (SEM and Light Microscopy)

Scanning electron micrographs of fibers were analyzed using ImageJ software (v1.54f; NIH, Bethesda, MD, USA), to determine fiber diameters and directionality. Polygons were drawn along and across fibers for straight distances of 0.5–11 µm (1–5 measures per fiber, averaging fiber diameter across 2.4–40 µm of each fiber’s length). This produced an average diameter for the demarcated short length of fiber, and these averages were in turn averaged per fiber.

Directionality analyses of SEM images produced similar results to light microscopy, but as light micrographs assessed larger areas, these were preferred for the presented data. Light micrographs from ×4, ×10, ×20, and ×40 objectives all generated similar data, and so all these were combined in the presented graphs.

### 2.9. Fluorescence Microscopy and Image Analysis, Cultures

Immunostained samples were imaged (fluorescence/phase contrast channels; Zeiss Axio Observer, z-stack microscope), and counterpart micrographs were merged using Zen software (microscope and software: Zeiss, Oberkochen, Germany) [36]. A minimum of three microscopic fields per culture were assessed for all conditions. In fluorescence micrographs (×20 objective), individual cells were manually delineated and then measured, providing morphometric data (ImageJ; n = 15–42) [37].

**Feret’s diameter:** Feret’s (maximum) diameter represents the furthest two points of each cell (caliper diameter). Feret’s minimum diameter indicates the shortest distance at which two parallel lines can restrict the entire cell [38].

**Aspect ratio (AR):** determined for each cell by dividing Feret’s maximum by Feret’s minimum; for a circle, this value is 1, and higher values indicate elongation of cells.

**Process length:** Lower magnification micrographs (×10 objective) were used for analysis of process length, as many elongated cells extended beyond the micrograph at higher magnification. For each of 4 microscopic fields, 10 randomly chosen processes were measured from the soma edge to the furthest extent of the process (freehand tool, ImageJ; n = 70–150). Tuj1^+^ cells were selected for analysis where a neurite could definitively be associated with 1 soma, ignoring dense clusters of Tuj1^+^ somata, which prevented accurate delineation of cells/neurites. It should be noted that astrocyte morphologies often lack fine processes, rendering some cells unsuitable for length analyses. However, Feret’s diameter is still applicable to these cells as an indicator of cell extension.

### 2.10. Directionality/Alignment Analysis

Directionality analyses were performed using micrographs of fibers alone and also for cultures (analyzing fluorescence channel alone for GFAP or Tuj1 vs. phase contrast alone for fibers). These analyses used the Directionality plugin for ImageJ software, as previously described, and generated histograms summarizing the range of fiber orientations present in each image [24]. A degree value is provided for the most frequently represented orientation of lines in the image, and this value for cells (e.g., GFAP) was subtracted from the counterpart value for fibers, such that small values were produced when cells were mostly aligned with the dominant fiber orientation.

### 2.11. Statistical Analyses

Each culture was established from a separate mouse litter (n = 3–4 biological replicates for each dataset). Data were analyzed using Prism statistical analysis software (GraphPad v6.07, Boston, MA, USA) and expressed as mean ± standard deviation (SD) unless declared otherwise. Unless otherwise stated, all data were analyzed using Kruskal–Wallis, with post-tests comparing data between substrates (at each time point), as well as comparing different time points for the same substrate.

## 3. Results

### 3.1. Neural Cell Responses to Nanofibers

#### 3.1.1. Astrocyte and Neuron Proportions Were Similar on Both Coverslips and Fibers

Neural co-cultures were successfully established on glass coverslips and fiber constructs. Immunostaining demonstrated the presence of GFAP^+^ astrocytes (25–35%) and Tuj1^+^ (BIII^+^) neurons (35–40%) in all cultures (n = 4) and across all substrates. A proportion of cells were GFAP^−^/Tuj1^−^ (25–30%), most likely a mixture of oligodendroglia and microglia [1].

#### 3.1.2. Astrocyte and Neuron Morphologies Differed on Coverslips and Fibers

**Figure 2:**
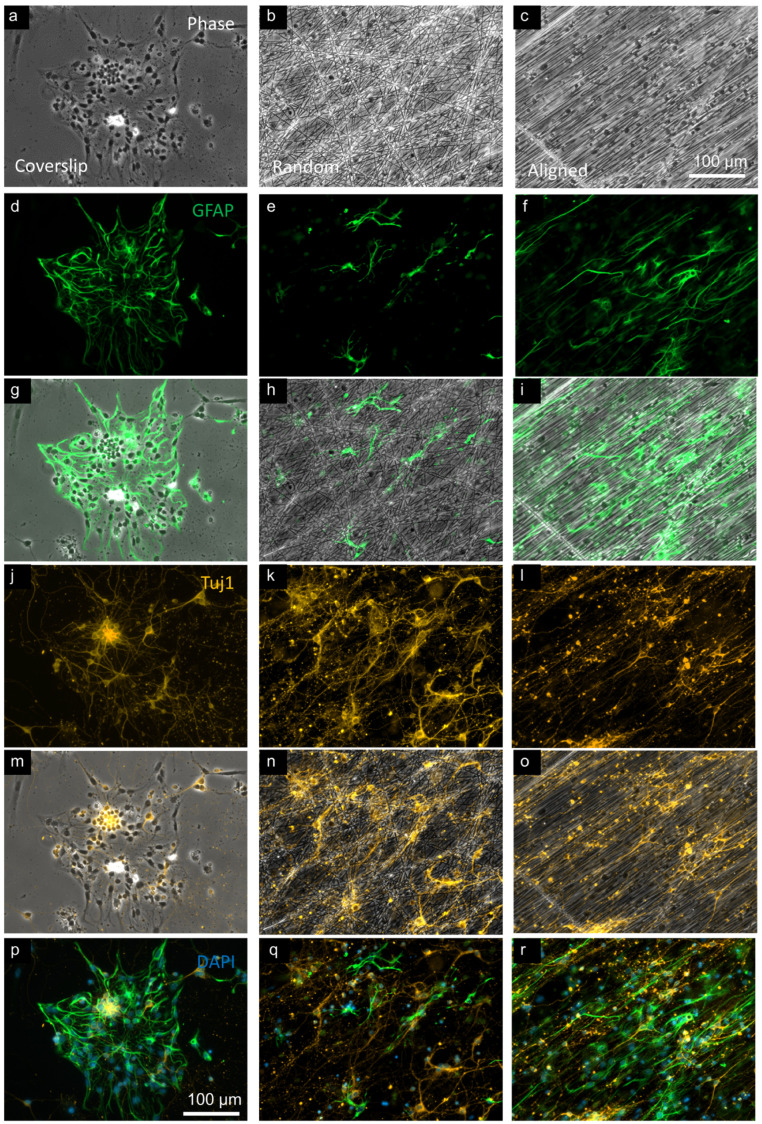
Fluorescence micrographs of neural co-cultures on glass and PCL fibers. (**a**–**c**) Phase contrast micrographs comparing neural co-cultures on coverslips, then randomly oriented fibers, and aligned fibers. Counterpart micrographs in each column then show GFAP (**d**–**f**), GFAP-phase merge to indicate astrocyte association with fibers (**g**–**i**), Tuj1 (**j**–**l**), Tuj1-phase merge to indicate neuronal association with fibers (**m**–**o**), and GFAP-Tuj1-DAPI merge (**p**–**r**) to show nuclei (DAPI^+^). GFAP: glial fibrillary acidic protein, astrocyte marker; PCL: polycaprolactone; Tuj1: neuronal marker.

On coverslips, astrocytes showed flattened morphologies, typical of in vitro culture, with variable size and shape. In random and aligned construct cultures, there was clear evidence of cell–fiber interaction, where cells displayed elongated processes, which often followed the path of identifiable nanofibers (Figure 2). In terms of cellular morphologies, GFAP^+^ cells were rarely bipolar on coverslips but were frequently observed to be bipolar in nanofiber cultures. Tuj1^+^ cells on coverslips also exhibited typical morphologies, with small somata, and fine (small diameter) processes, sometimes unipolar, but often with two or more processes. In fiber cultures, Tuj1^+^ cells exhibited similar morphologies, often with neurites in close association with PCL fibers.

In construct cultures, the majority of astrocytes and neurons showed interactions with fibers. Both GFAP^+^ and Tuj1^+^ cells frequently associated with individual fibers, often for long distances. Although cells were often adherent to a single fiber, some cells were observed adhering to multiple fibers, including at markedly different orientations (up to 90° from the original fiber). Various patterns of cell–fiber association were observed. Some cells were clearly associated with fibers for the entirety of the cell length, whereas other cells would show intermittent association with one or more fibers. Clustering of cells was more frequent on coverslips than on fibers.

#### 3.1.3. Astrocytes and Neurons Showed Elongated Morphologies on Nanofibers

Morphometric analyses were performed for GFAP^+^ and Tuj1^+^ cells, including Feret’s diameter (the distance between the furthest two points of a cell) and AR (caliper length divided by width). Feret’s diameter and AR are therefore indicators of the extent of elongation of an individual cell [38,39].

**Figure 3:**
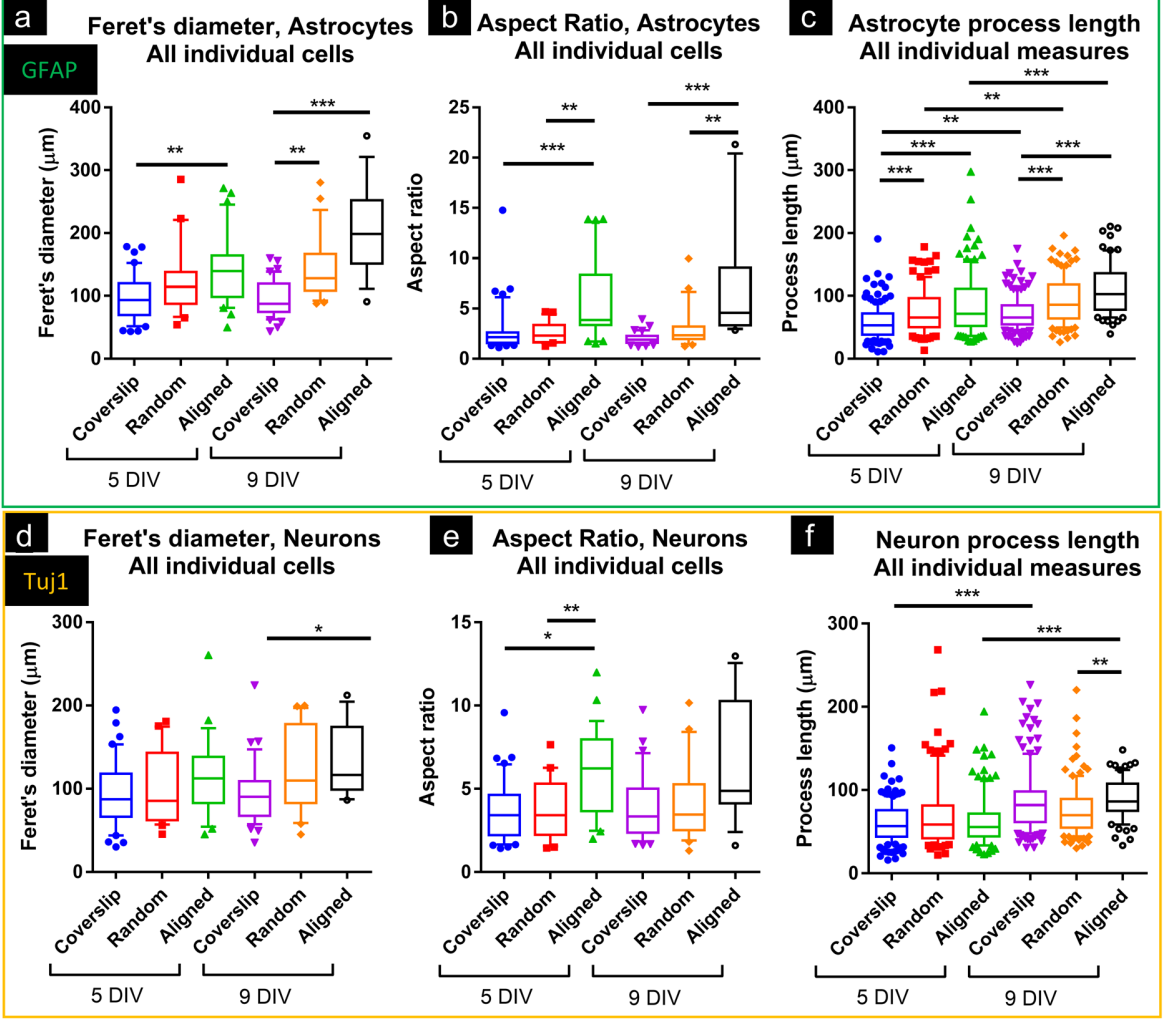
Astrocytes and neurons on PCL fibers exhibited elongated morphologies. Box and whisker (10–90%) plots showing Feret’s diameter, aspect ratio, and process length, for GFAP^+^ cells (green box) and Tuj1^+^ cells (orange box). (**a**) Astrocyte Feret’s diameter was greater on aligned constructs (5- and 9DIV vs. coverslips). At 9DIV, it was also greater on randomly oriented constructs vs. coverslips. (**b**) Astrocyte aspect ratio (length/width) was greater on aligned constructs (vs. coverslips and random; 5- and 9DIV). (**c**) Average astrocyte process length was greater on fibers (aligned vs. coverslips, random vs. coverslips; 5- and 9DIV). All substrates showed greater process length at 9DIV vs. 5DIV. (**d**) Neuronal Feret’s diameter was greater on aligned constructs at 9DIV (vs. coverslips), but otherwise no differences were detected. (**e**) Neuronal aspect ratio was greater on aligned constructs (vs. coverslips and random) at 5DIV, but not at 9DIV. Average aspect ratio did not increase over time for any substrate. (**f**) Average neurite length was greater at 9DIV (vs. 5DIV) for coverslips and aligned constructs. It was also greater on aligned constructs vs. random at 9DIV. All stats: Kruskal–Wallis with Dunn’s post-tests; * *p* < 0.05; ** *p* < 0.01; *** *p* < 0.001.

For GFAP^+^ cells at 5DIV, Feret’s diameter was similar for coverslip and random cultures but greater in cultures on aligned fibers (Figure 3a). At 9DIV, Feret’s diameter was greater in both random and aligned cultures vs. coverslips. GFAP^+^ cells had greater AR on both random and aligned constructs vs. coverslips at both 5- and 9DIV (Figure 3b), indicative of cellular elongation. Individual GFAP^+^ processes were longer on both random (5DIV, 75 ± 35; 9DIV, 93 ± 37 µm) and aligned constructs (85 ± 49; 110 ± 40 µm) vs. coverslips (58 ± 27; 72 ± 26 µm) (Figure 3c). For Tuj1^+^ cells, Feret’s diameter was similar across all substrates at 5DIV, but greater on aligned constructs at 9DIV (133 ± 43 µm) vs. coverslips (93 ± 37 µm; Figure 3d). At 5DIV, AR for Tuj1+ cells was greater on random and aligned constructs vs. coverslips, but no differences were noted at 9DIV (Figure 3e). Neurite length was similar across substrates at 5DIV, but at 9DIV aligned constructs showed greater length (90 ± 25 µm) vs. random (76 ± 32 µm; Figure 3f). With respect to time in culture, neurites were longer at 9DIV on coverslips (5DIV, 60 ± 23; vs. 9DIV, 88 ± 38 µm), and on aligned constructs (64 ± 31; vs. 90 ± 25 µm).

#### 3.1.4. Astrocytes and Neurons Demonstrated Coincident Orientation with Fibers

**Figure 4:**
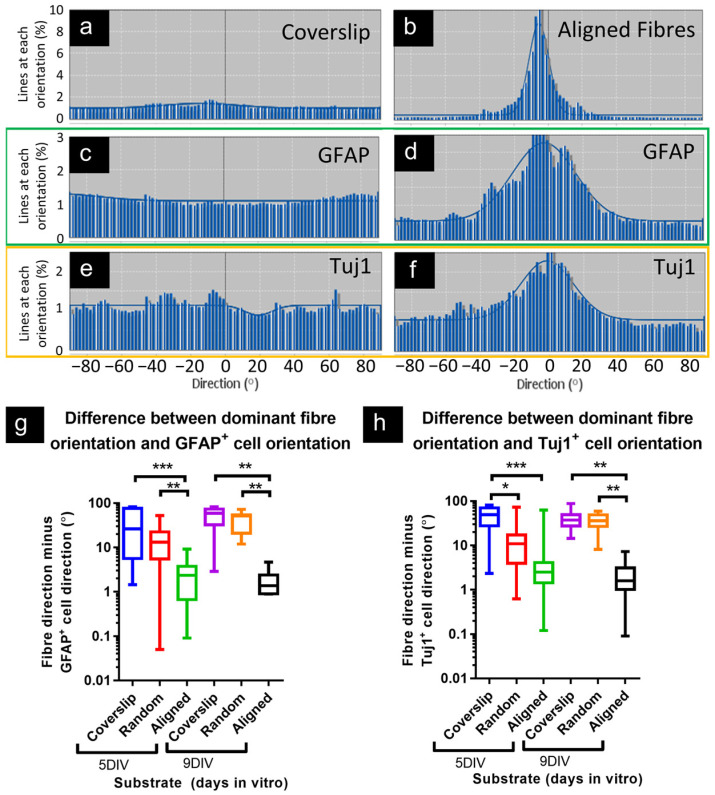
Astrocyte and neuron elongation matched the orientation of aligned fiber constructs. Directionality histograms show even distribution of orientations in coverslip cultures for (**a**) phase contrast, (**b**) GFAP, and (**c**) Tuj1 micrographs, consistent with cells extending in all directions approximately equally. However, strong peaks of orientation were present in histograms for aligned fiber constructs, with the peak in (**d**) phase contrast micrographs (fibers) matching the peaks in counterpart channels for (**e**) GFAP and (**f**) Tuj1. (**g**) Taking peak direction of orientation (°) for phase contrast micrographs, and subtracting the peak orientation for GFAP counterpart micrographs, showed smaller differences for aligned constructs vs. coverslips and vs. randomly oriented fibers, at both 5- and 9DIV. (**h**) Tuj1 micrographs more closely matched fiber orientation in aligned constructs at 5DIV (vs. coverslips) and at 9DIV (vs. coverslips and random). (**a**–**f**) are representative histograms from counterpart micrographs. (**g**,**h**) * *p* < 0.05; ** *p* < 0.01; *** *p* < 0.001; unpaired Kruskal–Wallis, Dunn’s post-test; n = 16, 16, 18, 10, 7, 5 individual image sets across cultures; plotted with log_10_ *y*-axis, due to the wide spread of data points.

Coverslips showed no strong peaks of orientation in phase or fluorescence channels, as would be predicted in the absence of any guidance cues (Figure 4). However, the strong peak orientation detected in aligned constructs (phase) was closely matched by the peak orientations in both green (GFAP) and red (Tuj1) fluorescence counterpart micrographs, indicating that cells preferentially elongated along the same axis, although with a broader spread of orientations overall, vs. phase micrographs (Figure 4a–f). Taking the single largest histogram value for phase and subtracting GFAP/Tuj1 equivalents produced small values (small differences in orientation) for aligned constructs vs. coverslips. Comparing this measure for all samples, there was greater disparity between the orientation of GFAP micrographs and phase micrographs for coverslips (disparity at 5- and 9DIV: 26° and 59°) and random constructs (13°, 51°) vs. aligned (2.3°, 1.4°; Figure 4g). This is consistent with the most common orientation of astrocytes closely matching the dominant orientation of the fibers in aligned constructs. The lack of coincidence between orientation in phase and Tuj1 micrographs from coverslips is illustrated in Figure 4h, vs. the similar phase-Tuj1 orientations calculated in aligned construct cultures (disparity at 5DIV, coverslip: 49°; aligned: 2.5°). At 9DIV, there was greater disparity between Tuj1 and phase for both coverslips (disparity at 9DIV: 37°) and random constructs (36°), versus aligned (1.6°). This is consistent with neuronal orientation closely matching fiber orientation in aligned constructs.

## 4. Discussion

These data demonstrate a ‘one-shot’ protocol for simultaneously deriving and interfacing mixed neuronal–glial cortical populations with a nanofiber scaffold, which induces simultaneous co-alignment of both astrocyte and neuron processes, offering organized and directional cell growth, in attempts to mimic in vivo cortical cell organization. PCL fibers induced dramatic morphological changes in both neural cell types, which could be reliably quantified using a directionality analysis method.

Both astrocytes and neurons showed process/cell elongation on aligned fiber constructs. Our observations are consistent with those of Reyes-Ramos et al., who reported breast cancer cell elongation on the same aligned fiber constructs, although without quantification of cell/process length [32]. In our study, many cells clearly associated with fibers, frequently following the path of identifiable individual fibers for distances of 200 µm, with the longest GFAP^+^ process reaching ~300 µm, and some neurites exceeding 200 µm (Figure 3). These fiber-associated cells adopted very narrow morphologies, with high aspect ratios (aligned, 5- and 9DIV: 3.8 and 4.5). These observations are in contrast to cultures on coverslips, where astrocytes typically have small aspect ratios (5- and 9DIV: 2.1 and 1.9), spreading their membrane in all directions.

For astrocytes, elongation of cells was consistent with the greatly increased Feret’s diameter and aspect ratio, as well as increased process length, with the latter also increasing over time (9DIV vs. 5DIV). Directionality analyses confirmed that software-detected lines in images of GFAP^+^ cells were largely oriented in the same direction as fibers, consistent with the longest axis of astrocytes being aligned with fibers. These astrocyte responses are similar to those reported by Lau et al. (2014), who cultured mouse forebrain astrocytes (P1.5) on PCL fibers (uncoated; diameters: random, 400 ± 110 nm; aligned, 450 ± 150 nm) [40]. GFAP^+^ cells on aligned scaffolds ‘sometimes exceeded 300 µm’, with processes at 12DIV of 63 ± 3 µm (random) and 90 ± 3 µm (aligned). AR was only reported as ≥2, but micrographs suggest greater minimum Feret’s diameters than observed in this study, consistent with astrocytes not associating as closely to individual fibers as the astrocytes in this study.

Xia et al. (2018) also compared astrocyte responses on randomly oriented and aligned poly(methyl methacrylate; PMMA) fibers [41]. Micrographs show some astrocytic alignment with fibers, suggested to increase between days 2 and 4 of culture. The longest cell process was the only morphological measure, reported at 4 days as PMMA film (non-nanofiber control) 99.7 ± 37.6, non-aligned fibers 73.2 ± 26.1, and aligned 122.5 ± 43.3 μm. Whereas Xia et al. reported only the *longest* cell processes, for rat astrocytes (P2 cortex), the current study reports the *average* process length for mouse astrocytes. At 5DIV, these were: coverslip 58 ± 27, random 75 ± 35, aligned 85 ± 49 (Figure 3).

In standard 2D cultures, neurons typically have larger aspect ratios than astrocytes, often showing unipolar or multipolar morphologies, with far finer processes than astrocytes. This standard morphology likely explains the more limited morphological differences observed for Tuj1^+^ cells. Neurite length was greater at 9DIV vs. 5DIV on coverslips and aligned constructs, indicating continuous extension over time, but aligned constructs did not show greater average length vs. coverslips at either timepoint. Directionality analysis showed neurons to be closely oriented with the dominant fiber orientation in aligned constructs, at both 5- and 9DIV, suggesting that fibers were directing neurite extension.

These observations of long, parallel cell processes are consistent with aspects of the cytoarchitecture of the developing cortex, particularly with reference to radial glial cells (RGCs), which serve as biological scaffolds, guiding migratory cells to appropriate cortical layers, where they differentiate and generate new tissue [42]. RGCs differentiate into astrocytes, similar to the morphologies demonstrated here on aligned nanofibers [43]. The parallel alignment of numerous neurites is also typical of the developing cortex [4].

Beyond the cortical cell alignment shown here, there may be several advantages from inducing neural cell elongation on implanted nanofiber scaffolds. A typical neuropathological feature is the glial scar, which consists of hypertrophic astrocytes (astrogliosis), ‘walling off’ lesions [44]. This severely limits cell migration beyond the scar, including for neurite extension [44,45]. Altering astrocyte morphology, for example, by inducing an elongated, narrower morphology, may serve to reduce this blocking function of the glial scar, enabling lesion access for cells/axons, etc. [46]. Lau et al. (2014) have suggested that PCL fibers induced a ‘cytotrophic’ (pro-repair) phenotype in primary astrocytes [40]. There are widely recognized therapeutic advantages to promote axonal re-growth across lesions, or otherwise guide (re)growing axons to a desired innervation site [45,47]. Nanofibers have shown elongation of neurites and directional guidance of neurites [16,48,49]. There are also indications that ECM-like signals (e.g., decorating fibers with molecules promoting cell adhesion or with trophic factors) promote cell migration/elongation [11,50,51]. Further, it is suggested that fiber-associated cells themselves may serve as cytotrophic guidance cues; e.g., astrocytes elongated on fibers may serve as a substrate promoting migration/elongation of other cells/neurites [46,52,53].

This protocol offers a relatively rapid and simple process to mimic several important features of cortical cytoarchitecture, but it does have limitations. Although the two-dimensional nature of these cultures renders them amenable to a wide range of analytical techniques, including live cell imaging, they do not replicate the three-dimensional nature of cortical tissue. There may be scope to address this by layering multiple sheets of cell-seeded fibers. Having established proof-of-concept for this culture system, future work will address the induction of specific neuropathological features.

## 5. Conclusions

This study describes a simplified, one-shot in vitro protocol for simultaneous derivation and nanofiber-induced alignment of a mixed population of primary cortical astrocytes and neurons on highly organized nanofiber meshes. Both astrocytes and neurons showed cell processes following fibers, with dramatic elongation of astrocytes induced. Aligned constructs induced directed orientation of neurons and astrocytes, which could be reliably quantified and assessed using our directionality analysis method for cell–fiber interactions. We consider our technically facile protocol offers an advanced method for inducing targeted and directional growth of complex populations of cortical neural cells in attempts to simulate cortical circuits in vitro. We consider the approach offers a versatile tool by which the cell-material interactions of multiple neural cell populations can be simultaneously evaluated using a brain mimetic neural cell culture method. This is relevant for modeling tissue development and neuropathology in vitro, and for testing therapeutic interventions.

## Data Availability

Data will be made available upon request.
